# Exploratory Longitudinal Associations Between SAPs and Individual and Contextual Variables: Toward the Development of Educational Guidelines

**DOI:** 10.3390/bs16091664

**Published:** 2026-09-16

**Authors:** Carmelo Francesco Meduri, Concettina Caparello, Maria Imbesi, Carolina Gonzálvez, Luana Sorrenti

**Affiliations:** 1 Department of Health Sciences, University Magna Graecia of Catanzaro, 88100 Catanzaro, Italy; carmelofrancesco.meduri@unicz.it (C.F.M.); maria.imbesi@studenti.unicz.it (M.I.); 2 Department of Classical, Linguistic and Educational Studies, Kore University of Enna, 94100 Enna, Italy; concettina.caparello@unikore.it; 3 Department of Developmental Psychology and Teaching, University of Alicante, 03690 Alicante, Spain; carolina.gonzalvez@ua.es; 4 Department of Clinical and Experimental Medicine, University of Messina, 98161 Messina, Italy

**Keywords:** school attendance problems, academic procrastination, school climate, perception of competences, mastery orientation, learned helplessness

## Abstract

School attendance problems (SAPs) are a widespread phenomenon with significant academic, psychological, social, and relational consequences. These issues are particularly prevalent among adolescents worldwide and require careful examination of the individual and contextual factors associated with their onset and persistence to develop effective prevention and intervention strategies. Grounded in an ecological and systemic framework of school absenteeism, this longitudinal study examined the reciprocal associations between SAPs, learned helplessness, mastery orientation, academic procrastination, perceived competence (general, academic, relational, leisure), and school climate (teacher support, peer connection, school connection, affirming diversity, clarity of rules, and reporting and seeking help) in a sample of 523 high school students (Mage at T1 = 15.64, SD = 0.97), using a cross-lagged panel model. Results indicate temporal stability of SAPs and the examined variables over one school year. Notably, alongside the longitudinal associations between SAPs and different individual and contextual variables, a significant bidirectional relationship emerges between learned helplessness and SAPs, suggesting that feelings of helplessness are linked to greater school disengagement and increased absences. This finding confirms the importance of emotional, motivational, and cognitive vulnerabilities in shaping school participation trajectories. The results emphasize the need for school-based prevention and intervention strategies targeting these factors. Accordingly, guidelines are proposed for educational interventions addressing SAPs.

## 1. Introduction

Regular school attendance is an important indicator of students’ educational functioning and psychosocial adjustment (Finn & Zimmer, 2012; Kearney & Graczyk, 2014; Sorrenti et al., 2024). It is associated with better academic outcomes, greater future employment opportunities, and a higher quality of life, while absenteeism and early school leaving are associated with negative educational, social, and professional outcomes (Credé et al., 2010; Nieuwoudt, 2020).

In this context, school attendance problems (SAPs) are getting more attention from the scientific community. SAPs is an umbrella construct that encompasses various manifestations of persistent difficulties in attending school, all of which have the potential to disrupt the continuity of a student’s educational journey (Heyne et al., 2019; Larsen et al., 2022). More broadly, the term SAPs is used to talk about the problems with keeping up with regular school attendance. This includes different methods and reasons for not going to school. This terminology reflects the multifaceted and heterogeneous nature of the phenomenon, including different manifestations, such as school refusal, truancy, etc.

A relevant model for understanding the complexity of SAPs among adolescent students, is proposed by Havik et al. (2015), who conceptualize SAPs as a multidimensional phenomenon and distinguish between legitimate and illegitimate absenteeism according to different underlying reasons. The model highlights how absenteeism can arise from a broad range of factors, not only related to the student’s desire to avoid school. In addition to absences due to illness or family emergencies, the authors also identify psychosomatic symptoms as possible indicators of school-related distress.

Moreover, according to Havik et al. (2015) and Havik and Ingul (2021), SAPs can be interpreted as the result of a complex interaction between psychological, relational, and motivational processes, which can significantly influence educational trajectories. From this perspective, numerous studies highlight the central role of the school as a fundamental ecological environment for individual development (Bronfenbrenner & Morris, 2007; Havik & Ingul, 2021). The systemic-ecological approach allows us to understand school attendance issues through an integrated model, in which risk factors and protective resources are considered within the reciprocal interactions between the individual and the context (Bronfenbrenner & Morris, 2007).

Among the individual factors, numerous studies have shown that both learned helplessness (LH) and academic procrastination can contribute to school absenteeism (Dan & Benovich, 2023; Sorrenti et al., 2016). LH refers to a condition in which individuals tend to perceive events as uncontrollable and failures as inevitable, attributing them to internal, stable, and unchangeable causes (Abramson et al., 1989; Seligman & Maier, 1967; Filippello et al., 2020). In educational contexts, such beliefs can result in a persistent sense of ineffectiveness, reduced motivation, and a progressive tendency to avoid schoolwork (Weiner, 1986). Students with high levels of LH tend to avoid tasks perceived as challenging, preferring simpler activities to reduce the risk of failure, which is interpreted as further confirmation of their own inability (Raufelder et al., 2017). Consequently, they place little value on academic effort, believing their ability to influence learning processes and improve academic performance to be limited (Sorrenti et al., 2016; Pérez-Marco et al., 2025). In line with this interpretation, Sutherland et al. (2004) emphasize that repeated experiences of academic failure can gradually lead students to doubt their intellectual abilities and their ability to overcome the difficulties they encounter. This belief leads to a reduction in efforts aimed at achieving academic goals, fueling a vicious cycle of further failures that further reinforces the perception of helplessness and inadequacy.

LH not only undermines motivation but can also lead to the adoption of dysfunctional strategies in response to educational demands. Of these, academic procrastination, defined as the deliberate postponement of school assignments and activities despite awareness of the possible negative consequences, is particularly significant (Steel, 2007). Students who perceive little control over their own performance tend to postpone completing tasks as an avoidance strategy, to reduce the emotional distress associated with the possibility of failure and performance anxiety, leading to a progressive disengagement from school (Sorrenti et al., 2016; Niknam et al., 2023).

Alongside the vulnerability factors, the literature has identified motivational resources capable of promoting school attendance. Among these, Mastery Orientation (MO), a behavioral pattern characterized by seeking out challenging tasks, persevering despite difficulties, and maintaining positive expectations of success even after failures (Dickhäuser et al., 2011). MO students, who view learning process as an opportunity for personal growth and skill development, tend to perceive themselves as more competent and exhibit higher levels of self-efficacy (Filippello et al., 2013; Losier et al., 1993). These characteristics result associated with greater satisfaction with the overall school experience, as well as higher levels of engagement and participation in school activities (Ames & Archer, 1988; Green et al., 2012). From this perspective, MO may represent an important protective factor against SAPs (Sorrenti et al., 2018).

Regarding contextual factors, a crucial role is played by school climate, defined as the quality of the school environment as perceived by students in terms of relationships, support, safety, and school organization (Kearney et al., 2023a; Melvin et al., 2019). The perception of a positive school climate, characterized by supportive relationships with teachers and peers, a sense of belonging, safety, and clear rules, promotes the fulfillment of psychological needs, contributing to students’ engagement in school life (Hamlin, 2021). Conversely, school climate perceived as hostile or unsupportive can compromise school adjustment and increase the risk of disengagement, school avoidance, and absenteeism (Heyne et al., 2019; Kearney et al., 2023b).

The constructs described so far exhibit a dynamic trajectory over time, with effects emerging in the short and long term. Prolonged school non-attendance represents a relevant risk factor associated with adverse outcomes across lifespan. During adolescence, school absenteeism is linked to poor school performance and a higher risk of engaging in risk behaviors, leading to an increased probability of early school leaving (Hawkrigg & Payne, 2014). In the long term, SAPs are associated with persistent socioeconomic and mental health disadvantages in adulthood. Lower levels of education are, in fact, correlated with less job stability, a reduced sense of personal control, and lower social support, factors that contribute to greater psychological vulnerability and an increased risk of internalizing disorders, including depressive symptoms and adjustment disorders (Allison et al., 2019). Similarly, LH influences overall functioning, affecting academic performance, physical and mental health, and social well-being. Specifically, it has been shown that the persistence of LH can lead to a decreased initiative and interest in pleasurable activities, or anhedonia, both symptoms typically observed in depression (Tafet & Ortiz Alonso, 2025). Moreover, LH leads individuals to perceive consequences as independent of their own actions, resulting in a negative impact on future behavior, such as a lack of willingness to try to improve their abilities (Winterflood & Climie, 2020). Academic procrastination, on the other hand, has divergent effects in the short and long term. Tice and Baumeister (1997) highlight that intentional postponement of tasks can initially lead to a reduction in perceived stress, as it allows individuals to temporarily avoid activities perceived as unpleasant or demanding. This benefit, however, is only apparent and short-lived. As deadlines approach, the accumulation of uncompleted tasks leads to increased time pressure and cognitive load, resulting in increased stress and poorer performance. From this perspective, procrastination is a dysfunctional behavior in which an immediate emotional benefit is gradually overshadowed by negative medium- to long-term consequences.

Considering these findings, SAPs cannot be interpreted exclusively as the result of individual difficulties but must be understood within a dynamic system of reciprocal interactions among individual, relational, and contextual factors. An integrated examination of these variables could allow for a deeper understanding of the processes that can either promote or hinder students’ school engagement. Moreover, despite growing evidence of associations between these variables, our understanding of the longitudinal and reciprocal processes that shape their development over time remains limited. Few studies have simultaneously analyzed the directions of influence between SAPs and psychological and academic factors within integrated longitudinal models.

Therefore, the aim of this study is to explore the longitudinal relationships and mutual associations between SAP, psychological and academic factors, and school climate. This will help to outline useful educational guidelines for addressing this phenomenon.

## 2. Present Study

School attendance problems (SAPs) represent a complex and multifaceted phenomenon associated with significant school, psychological, and social consequences. Considering that rates of SAPs have increased worldwide over the last decade (UNESCO, 2020; Ydo, 2020), it is essential to investigate the key factors associated with the onset and persistence of these difficulties (Kearney, 2008; Kearney et al., 2004), in order to identify educational guidelines and targeted interventions aimed at effectively preventing and addressing the phenomenon (Pérez-Marco et al., 2025).

In Italy, SAPs are an important educational concern and are closely related to the broader phenomenon of school dropout. Persistent attendance difficulties can increase the risk of students leaving school early. Italy has historically recorded higher early school leaving rates than the European average, but the most recent data show a substantial decline, from 14.2% in 2020 to 8.2% in 2025 (Ministero dell’Istruzione e del Merito, 2026). This decline may reflect the impact of policies to combat the phenomenon, but attendance challenges remain a significant issue, requiring ongoing attention to students’ participation (Di Martino et al., 2026).

Numerous studies have highlighted the negative short- and long-term impact of absenteeism problems (Bianchi et al., 2022; Gubbels et al., 2019). SAPs have been associated with poorer academic performance, difficulties in social and emotional adjustment, and multiple maladaptive outcomes over time, especially in the absence of timely and effective interventions (Ansari & Pianta, 2019; Gottfried, 2011). Grounding on the categorical model of school absenteeism proposed by Havik et al. (2015), which examines the different underlying reasons of attendance problems from ecological and systemic perspectives, the aim of this study is to identify the factors associated with SAPs over time and to provide educational guidelines for their prevention and management.

In recent years, the literature has highlighted a wide range of individual and contextual factors associated with SAPs (Daraganova, 2014; Kearney et al., 2023a; Sorrenti et al., 2024). Emotional, motivational and self-regulatory vulnerabilities, such as LH and academic procrastination, have particularly emerged as important individual factors associated with lower levels of school involvement and greater avoidance behaviors (Balfanz et al., 2007; Meduri et al., 2026b; Pérez-Marco et al., 2025; Sorrenti et al., 2016). Conversely, MO, perceived competence, and a positive school climate have been found to be positively associated with higher levels of adaptation and greater participation in school activities (Daily et al., 2020; Hendron & Kearney, 2016; Meduri et al., 2026a).

To our knowledge, no previous longitudinal study has examined all the constructs included in this model simultaneously within a single cross-lagged framework. Consistent with the ecological model (Bronfenbrenner, 1994), SAPs can be conceptualized as resulting from the dynamic interaction between individual psychological processes and contextual factors related to school and interpersonal relationships (Guz & Irsheid, 2024; Havik et al., 2015; Kearney et al., 2023b; Middleton et al., 2025). The present study adopts an exploratory cross-lagged panel design to investigate potential reciprocal associations between SAPs and a range of individual and contextual variables in high school students.

Accordingly, the study focuses primarily on individual psychological and motivational processes and contextual factors within the school setting. These factors are viewed as interconnected components of a broader ecological system, with the ecological perspective providing the overarching framework for understanding the multifaceted nature of SAPs. In addition, from a positive psychology perspective, perceived competence and MO can be considered personal resources that support students in adapting to school demands, engaging with their studies, and coping with the challenges they face (Linnenbrink & Pintrich, 2002; Sorrenti et al., 2018). At the same time, the interpretation of attendance-related behaviors is informed from a behavioral perspective. According to this perspective, school attendance difficulties can be understood in relation to the functions and contextual contingencies that may contribute to the maintenance of these behaviors (Kearney & Silverman, 1993).

Specifically, the study will examine the processes involved in the persistence and development of SAPs over time. The aim is to develop guidelines for educational interventions that prevent and reduce these issues by addressing the emotional, cognitive, and behavioral processes related to school attendance difficulties.

The first objective is to investigate the relationships between SAPs, LH, MO, academic procrastination, perceived competencies and school climate at Time 1 and Time 2, as well as the temporal stability of these variables using autoregressive effects.

The second objective is to examine the reciprocal longitudinal associations between SAPs and the individual and contextual factors under consideration using cross-lagged effects.

The third objective is to provide guidelines for educational programs to address SAPs based on the longitudinal associations that have emerged, interpreted considering the existing literature.

## 3. Methods

### 3.1. Participants

The longitudinal study involved 523 high school students from southern Italy who were in their second, third, fourth or fifth year of study and took part in both data collection phases (T1 and T2). The two assessment periods (T1 and T2) took place one school year apart, with T2 occurring approximately 12 months after T1. Of these participants, 343 (65.6%) were male, 179 (34.2%) were female and one (0.2%) did not indicate their gender. The mean age of participants was 15.64 years (SD = 0.97) at T1 and 16.41 years (SD = 1.18) at T2. Additionally, 514 participants were Italian and nine were non-Italian. On a scale of 1 to 10, where higher scores indicate better academic performance, the average grade was 7.30 (SD = 0.84) at T1 and 7.39 (SD = 0.87) at T2. The average number of school absences was 23.1 (SD = 11.9) at T1 and 23.3 (SD = 11.0) at T2. Students with disabilities or special educational needs did not participate in the study.

### 3.2. Instruments

The first part of the survey involved a demographic questionnaire, which was designed to collect information on the age, gender and nationality of the sample, as well as their educational background (e.g., number of absences and grade point average).

SAPs were assessed using the Italian validation of the Reasons for School Non-Attendance Scale (Sorrenti et al., 2026; Havik et al., 2015). The ARSNA is a self-report questionnaire consisting of 13 items rated on a 4-point Likert scale ranging from 0 (“never”) to 3 (“quite often”), designed to assess the frequency with which students were absent from school during the previous three months for specific reasons. Each item is introduced by the stem: “How many times have you been absent from school during the past three months because…”. The instrument demonstrated adequate internal consistency at both time points (Cronbach’s α = 0.80 at T1; Cronbach’s α = 0.81 at T2).

LH and MO were assessed using the Learning Helplessness Questionnaire (LHQ; Sorrenti et al., 2015b), a self-report questionnaire consisting of 13 items divided into two subscales: Learned Helplessness (LH; 6 items; e.g., “When you can’t study a part of a subject, you get discouraged, thinking you won’t be able to pass the exam”) and Mastery Orientation (MO; 7 items; e.g., “You strive to study well for your own personal satisfaction rather than to obtain a high grade or a reward”). Items are rated on a 5-point Likert scale ranging from 1 (“not true”) to 5 (“very true”). Both subscales showed adequate internal consistency at both time points (LH: Cronbach’s α = 0.85 at T1 and 0.83 at T2; MO: Cronbach’s α = 0.73 at T1 and 0.76 at T2).

Academic procrastination was assessed using the short version of the Academic Procrastination Scale (APS-S; Yockey, 2016). The scale measures the tendency to postpone school activities through 5 items (e.g., “When I am assigned an assignment, I tend to put it aside and forget about it until the deadline is very close”), rated on a 5-point Likert scale ranging from 1 (“strongly agree”) to 5 (“strongly disagree”). The APS-S showed adequate internal consistency across both measurement occasions (Cronbach’s α = 0.88 at T1 and 0.87 at T2).

Students’ perception of competence in different life domains was assessed using the Perception of Competence in Life Domains Scale (PCLDS; Losier et al., 1993). The instrument consists of 16 items organized into four subscales (4 items each): perceived General Competence (e.g., “Sometimes I think I am not competent in many of the activities I participate in”), perceived Academic Competence (e.g., “In general, I believe I am a good student”), perceived Competence in Interpersonal Relationships (e.g., “I have many friends”), and perceived Leisure Competence (e.g., “I never seem to be able to effectively manage my leisure activities”). In the present study, the subscales showed adequate internal consistency at both time points: General Competence (Cronbach’s α = 0.81 at T1; 0.82 at T2), Academic Competence (α = 0.81 at T1; 0.81 at T2), Competence in Interpersonal Relationships (α = 0.81 at T1; 0.82 at T2), and Competence in Leisure time (α = 0.68 at T1; 0.67 at T2).

School climate was assessed using the What’s Happening In This School? questionnaire (WHITS; Aldridge & Ala’i, 2013), consisting of 48 items reflecting six fundamental dimensions of students’ perceptions of the school context: Teacher Support (TS; 8 items; e.g., “In this school, teachers try to understand my problems”), Peer Connection (PC; 8 items; e.g., “In this school, I feel accepted by other students”), School Connection (SC; 8 items; e.g.,: “I feel welcomed in this school”), Affirming Diversity (AD; 8 items; e.g.,: “In this school my cultural context is respected by the students”), Clarity of Rules (RC; 8 items; e.g.,: “In this school, school rules help me feel safe”), and Reporting and Seeking Help (RSH; 8 items; for example: “I can report episodes without anyone else knowing”). Responses were given on a 5-point Likert scale, from 1 (“almost never”) to 5 (“almost always”). All WHITS subscales demonstrated adequate internal consistency across both measurement occasions (TS: Cronbach’s α = 0.88 at T1 and 0.88 at T2; PC: α = 0.86 at T1 and 0.85 at T2; SC: α = 0.88 at T1 and 0.84 at T2; AD: α = 0.87 at T1 and. 86 at T2; RC: α = 0.88 at T1 and 0.87 at T2; RSH: α = 0.92 at T1 and 0.90 at T2).

### 3.3. Procedure

In accordance with the Code of Ethics of the Italian Psychological Association (AIP) and the 2013 Declaration of Helsinki, the study protocol was approved by the relevant ethics committee (UA-2023-06-29-4, University of Alicante). Students were informed about the objectives and methods of the research and provided written informed consent. For underage participants, this was obtained from parents; for students of legal age, it was obtained directly from the students themselves. They were also informed that participation was voluntary, that data would be collected anonymously and treated confidentially, and that they could withdraw from the study at any time without any consequences.

Data collection took place during school hours, with researchers administering questionnaires in the classroom. In both waves, completion of the questionnaires required approximately 30–40 min, and participants had the opportunity to ask questions throughout the procedure. Data were collected at two time points over a 12-month period, corresponding to one school year. The first survey (T1) was conducted between March and April 2025 and involved 800 students. The second survey (T2) took place between March and April 2026, with 523 students participating.

Considering the typical challenges associated with school-based longitudinal research, such as absences, transfers, and dropouts, the reduction in sample size between T1 and T2 is consistent with previous reports (Bianchi et al., 2022; Henneberger et al., 2023). In addition, this time interval is consistent with prior longitudinal studies on school absenteeism, which have shown that changes in school and socio-emotional adjustment processes can be observed over both short- and longer-term periods (Bianchi et al., 2022). The present study therefore focused on participants who completed both waves of data collection.

### 3.4. Data Analysis

All analyses were conducted using Jamovi (Version 2.3.28; The Jamovi Project, 2024). First, preliminary descriptive and correlational analyses were performed. Specifically, means, standard deviations, and Pearson correlation coefficients were calculated to examine the distribution of the variables and their associations at each measurement time point. Internal consistency coefficients (Cronbach’s alpha) were computed for all scales. Scale and subscale scores were computed as the mean of their respective items and used as manifest variables in all analyses.

A longitudinal analysis was conducted using a cross-lagged panel model to examine bidirectional relationships between SAPs and the individual and contextual variables under consideration. This approach, implemented within a structural equation modelling framework, allows for the examination of reciprocal associations between variables across time (Finkel, 1995; Little, 2024; Lucas, 2023; Selig & Little, 2012).

The model estimated autoregressive paths to account for the temporal stability of each variable over time, as well as correlations among all variables within each measurement wave. Cross-lagged paths were specified from SAPs at T1 to each individual and contextual variable at T2, and reciprocally from each individual and contextual variable at T1 to SAPs at T2, to examine bidirectional longitudinal associations among the constructs.

Model estimation was performed using the robust maximum likelihood estimator (MLR). Model fit was evaluated using standard fit indices, including the Comparative Fit Index (CFI) and the Tucker–Lewis Index (TLI), with values ≥ 0.95 indicating good model fit; the Root Mean Square Error of Approximation (RMSEA), with values < 0.08 indicating acceptable model fit (Kline, 2015); and the chi-square statistic (χ^2^).

## 4. Results

### 4.1. Descriptive and Correlational Analyses

Overall, the study variables (Table 1) were approximately normally distributed, with skewness and kurtosis values within acceptable thresholds (|skewness| and |kurtosis| ≤ ±2; Tabachnick et al., 2007). Table 2 shows the results of the correlation analysis of the study variables at Time 1 and Time 2. Correlation analyses indicated significant associations among SAPs, LH, MO, procrastination, perceived competences, and school climate dimensions at both time points. Specifically, SAPs were positively associated with LH and procrastination, and negatively associated with MO, perceived competences, and positive perceptions of the school climate at both Time 1 and Time 2. Overall, the pattern of correlations remained relatively stable over time, suggesting moderate to strong stability of the constructs across the one-year period.

### 4.2. Cross Lagged Model

**Figure 1:**
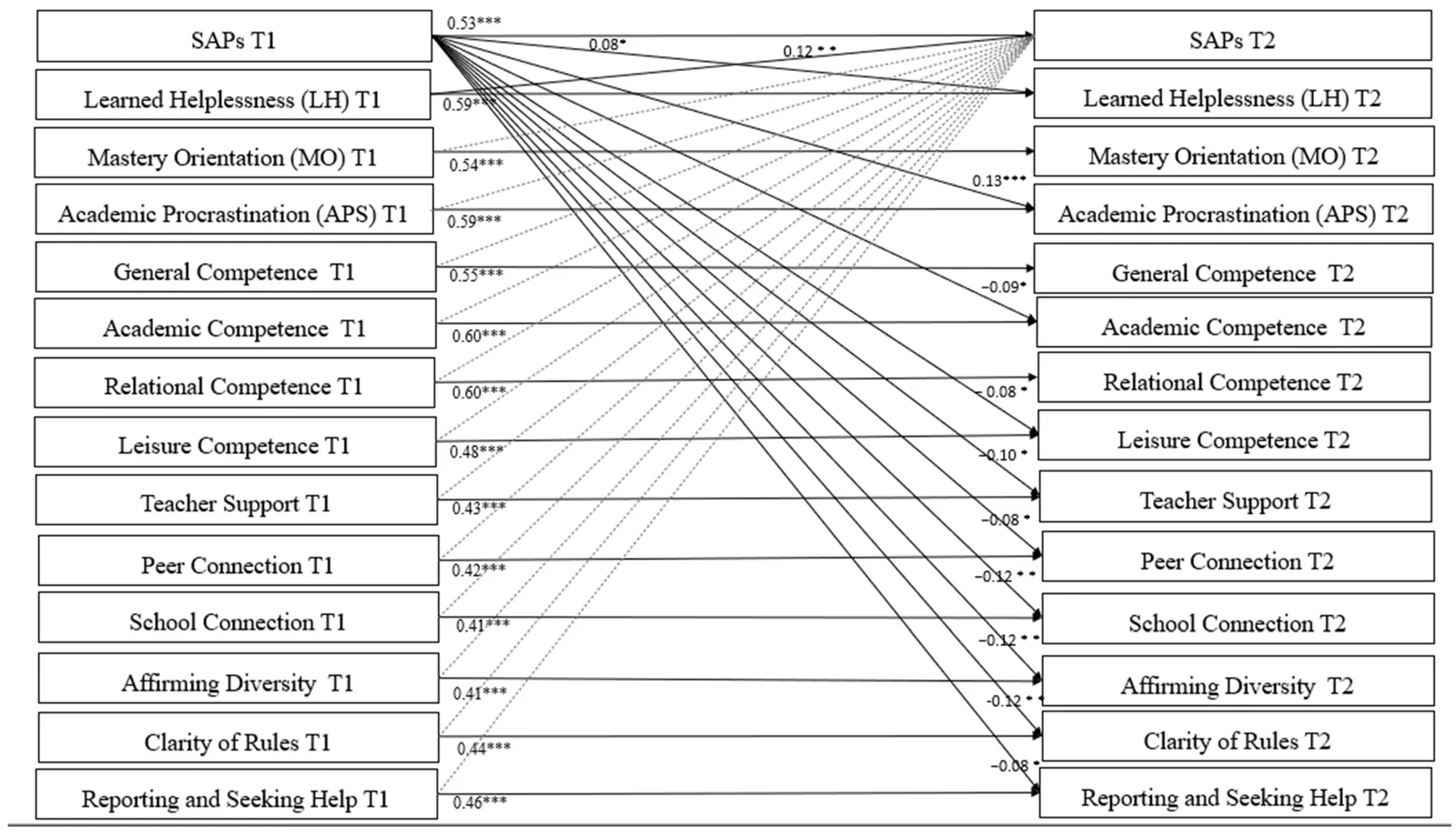
Cross-lagged model. Note. Values reported are standardized path coefficients (*β*). *** *p* ≤ 0.001, ** *p* ≤ 0.01, * *p* ≤ 0.05. Dashed arrows represent hypothesized bidirectional associations that did not reach statistical significance.

The results indicated that the model showed an acceptable fit to the data, χ^2^(156) = 437, *p* < 0.001, χ^2^/df = 2.80, RMSEA = 0.06, 95% CI [0.052, 0.065], CFI = 0.97, TLI = 0.92, and SRMR = 0.06. In line with our objectives, all autoregressive paths were significant (*p* < 0.001), indicating substantial temporal stability of the SAPs and the individual and contextual variables over the one-year period. Standardized autoregressive coefficients (Figure 1) ranged from β = 0.41 to β = 0.60, indicating good longitudinal stability of the constructs examined over time.

Regarding the cross-lagged effects, LH at T1 positively predicted SAPs at T2 (b = 0.06, *p* = 0.007, 95% CI [0.01, 0.10], β = 0.12). Conversely, SAPs at T1 positively predicted both LH (b = 0.14, *p* = 0.034, 95% CI [0.01, 0.28], β = 0.08) and APS at T2 (b = 0.28, *p* < 0.001, 95% CI [0.13, 0.43], β = 0.13). In addition, higher SAPs at T1 negatively predicted perceived academic competence (b = −0.22, *p* = 0.015, 95% CI [−0.40, −0.04], β = −0.09), leisure competence (b = −0.18, *p* = 0.039, 95% CI [−0.36, −0.09], β = −0.08), and several dimensions of school climate at T2, including teacher support (TS; b = −0.17, *p* = 0.014, 95% CI [−0.30, −0.03], β = −0.10), peer connection (PC; b = −0.13, *p* = 0.052, 95% CI [−0.26, 0.00], β = −0.08), school connection (SC; b = −0.20, *p* = 0.002, 95% CI [−0.32, −0.07], β = −0.12), affirming diversity (AD; b = −0.20, *p* = 0.003, 95% CI [−0.34, −0.07], β = −0.12), clarity of rules (RC; b = −0.20, *p* = 0.002, 95% CI [−0.33, −0.07], β = −0.12), and reporting and seeking help (RHS; b = −0.16, *p* = 0.036, 95% CI [−0.30, −0.01], β = −0.08).

### 4.3. Implications for Educational Programs

In line with the existing literature, the longitudinal results provided valuable insights for developing educational guidelines to prevent and combat SAPs. These findings have important educational implications that should be considered within the broader ecological framework proposed by Bronfenbrenner (1994, 2005). According to this framework, school absenteeism arise from the interaction between individual and contextual factors.

Integrating the associations that emerged in the study with the AGIA (2022) recommendations and the evidence from the analysis of the Erasmus+ SOS-Attendance project protocols (Caparello et al., 2025) allowed us to identify educational implications at individual and contextual levels.

At an individual level, the results emphasised the importance of early monitoring of psychological and motivational vulnerabilities, particularly LH as a potential risk indicator. In the context of this issue, we have seen the importance of promoting supportive, safe and collaborative school environments, as well as the development of teachers’ professional skills for the early identification of signs of SAPs.

## 5. Discussion

### 5.1. Temporal Stability and Longitudinal Associations Between SAPs, Individual Factors, and School Climate

School attendance has important short- and long-term consequences, influencing students’ personal, academic and professional growth (Credé et al., 2010; Cecchi, 2013; Daraganova, 2014). School, as a fundamental setting for cognitive, emotional, and social growth, enables students to interact with teachers and peers, engage in the exchange of thoughts and opinions, and participate in various educational activities (Kearney & Graczyk, 2014). Students may react differently to the school demands and different experiences proposed by an educational setting. They can successfully address and master educational challenges or develop feelings of ineffectiveness and helplessness (Seligman, 1975; Sorrenti et al., 2015b). The latter can lead to avoidance behaviors and the gradual postponement of schoolwork (Yockey, 2016). Together with the extra-school context, these experiences progressively contribute to students’ development of a sense of competence, influencing how they perceive their ability to deal with different developmental challenges (Deptuła & Borucka, 2020; Losier et al., 1993). Using a cross-lagged panel model, the results showed that SAPs, while remaining relatively stable over time, are associated with the various psychological and school dimensions examined longitudinally. Students with greater school attendance difficulties tend to exhibit increased motivational vulnerabilities, such as LH and academic procrastination, accompanied by a reduction in perceived competencies (both school and related to leisure time) and a progressively more negative perception of the school climate across its various dimensions. In line with the literature (Kearney, 2008; Meduri et al., 2026a), these students tend to gradually disengage from academic activities they perceive as frustrating or excessively difficult, developing expectations of failure and fears regarding their own abilities. Such experiences, by fostering feelings of helplessness and a sense of failure (Sorrenti et al., 2016, 2015a), support the procrastination of tasks (Ferrari et al., 1995; Steel, 2007). Concurrently, bidirectional analyses of the model have identified LH as a significant antecedent of SAPs, suggesting that early vulnerability in emotional, motivational, and cognitive processes regarding educational activities may contribute to the onset of SAPs. In this sense, feelings of helplessness, the perception of having little control over events, and the resulting disengagement from school are factors that predict SAPs (Cheung & Pomerantz, 2011; Kearney, 2008; Seligman, 1975). Overall, these findings suggest the presence of a potential reciprocal process between SAPs and LH, whereby feelings of helplessness are associated with higher levels of school disengagement and increased absences (Crown, 2024; Swargiary, 2025). Conversely, reduced attendance and participation in educational activities could perpetuate or exacerbate feelings of ineffectiveness and helplessness. From this perspective, progressive separation from school may represent not only a possible consequence of initial difficulties, but also a condition associated with their persistence over time, through a process of mutual reinforcement between the considered variables.

No significant longitudinal associations were found between SAP and MO. This suggests that this motivational dimension probably depends more on stable personal factors, such as learning goals and beliefs about one’s abilities, as well as school attendance experiences (Nerstad et al., 2016; Sorrenti et al., 2016).

Furthermore, our findings, consistent with previous studies (Havik et al., 2015; Thomas, 2019), highlight that SAPs are associated with a student’s negative perception of the school climate. In particular, due to reduced school participation, disengagement, and limited opportunities for socialization, students with these problems tend to interact less with teachers and peers, receive less academic support, and experience greater social isolation (Hendron & Kearney, 2016; Havik et al., 2015). This situation frequently contributes to a reduced sense of school connectedness and fosters the perception of school as a less equitable and inclusive environment, characterized by lower adherence to rules (Cameron et al., 2025; Heyne et al., 2019; Kearney et al., 2023a; Sorrenti et al., 2024).

In this context, SAPs have also been found to be associated with a negative perception of competence in both school and leisure activities over time. This finding is consistent with previous research, indicating that students with attendance difficulties tend to report lower levels of self-efficacy and perceived school competence (Kearney & Graczyk, 2014; Pérez-Marco et al., 2025). From this perspective, progressive disengagement from the school environment can reduce opportunities for successful experiences and active participation, thereby limiting the chance to develop and consolidate skills, and negatively influencing the perception of one’s abilities, even in leisure activities. In contrast, no significant relationships emerge between SAPs and the perception of relational and general competencies. This may be because our sample consists of adolescents who are at a stage in life where the social sphere is becoming increasingly important, and where interpersonal skills are heavily influenced by peer groups. During adolescence in particular, interpersonal skills and daily life development are strongly influenced by experiences gained outside of school, which significantly contribute to social and personal development. At this stage, relationships with peers outside of school are one of the main contexts through which adolescents develop their sense of self and their skills (Mahoney et al., 2009).

### 5.2. Educational Guidelines for the Prevention and Intervention of SAPs

In Italy, school absenteeism rates remain particularly high, especially in the southern regions, where approximately one in five young people leaves school prematurely, with more pronounced spikes during the transition between school cycles (AGIA, 2022). To address this issue, the Italian Authority for Children and Adolescents (AGIA, 2022) reviewed several intervention protocols implemented nationwide, identifying two core directions for structural measures: (a) transforming life and learning contexts while promoting early prevention, and (b) supporting targeted, multifaceted promotional and preventive actions designed to meet the needs not only of individual schools, but also of the wider community and of students themselves. The findings of the present study, interpreted in light of these recommendations and Bronfenbrenner’s ecological framework (Bronfenbrenner, 1994, 2005), and structured around the strengths and weaknesses of the Italian intervention protocols examined during the Erasmus+ SOS-Attendance program (Caparello et al., 2025), provide a solid conceptual foundation for developing educational guidelines that school staff and other stakeholders can use to prevent and address absenteeism in Italian upper secondary schools, while also promoting actions that foster greater student engagement, reinforce the importance of regular school attendance, and enable early intervention before the problem arises.

Given the multifaceted and context-dependent nature of SAPs, these guidelines should be regarded as a series of educational recommendations that can be adapted to meet students’ individual needs and the specific characteristics of their family, classroom and school environments.

Considering that the effectiveness of interventions aimed at preventing absenteeism must be interpreted within the broader, multilevel context in which this phenomenon occurs and in light of the factors that contribute to its development and persistence (Li et al., 2026), a first implication of the present study concerns the recognition of the warning signs of early school leaving, even before absences become detectable in official records and subsequently develop into chronic absenteeism (Kearney et al., 2023b). The emotional and motivational factors identified in this study—learned helplessness, along with a gradual decline in perceived competence—constitute, in line with previous studies (Pérez-Marco et al., 2026a; Piscitello et al., 2022), early indicators of this process. In particular, LH emerges as the vulnerability factor that best predicts SAPs in the medium to long term. These findings, consistent with previous studies (Meduri et al., 2026a), suggest that LH is not merely a factor correlated with school absenteeism, but a fundamental psychological condition that precedes it and underlies it. Consequently, it can be identified while students are still actively attending school. Educational guidelines should therefore consider LH as an independent early warning indicator of the risk of school absenteeism, well before chronic absenteeism becomes evident. In practice, the identification of these emotional and motivational vulnerabilities cannot rely solely on informal observations by teachers and families, nor should it be a reactive measure triggered by a decline in attendance (Kearney et al., 2023a; Li et al., 2026; Sletten et al., 2023). For this reason, in a context where educational monitoring has become a routine procedure within evidence-based policies and practices in the education sector, schools should implement systematic and periodic screenings to assess their students’ sense of control and perceived academic self-efficacy, addressing these underlying beliefs before they lead to early school leaving (Sälzer et al., 2024). From this perspective, schools should establish clear screening protocols under which high levels of LH trigger targeted support—such as psychological counseling or personalized tutoring—regardless of attendance rates. Doing so would realize the true potential of preventive intervention, which lies precisely in the gap between psychological vulnerability and behavioral manifestation, allowing professionals to act while students are still attending school, rather than after absenteeism has already taken root (Kearney et al., 2023a; Li et al., 2026). Consequently, investing in systematic screening tools enables teachers, counselors, and psychologists to intervene proactively, addressing disengagement at its root rather than merely reacting to its symptoms.

However, from an ecological perspective, equal attention must be paid to the context in which students learn and grow (Piscitello et al., 2022). The school environment can exacerbate the experience of LH by influencing students’ sense of competence in its relational, organizational, and instructional dimensions (Kim et al., 2023; Raufelder & Kulakow, 2022) thereby increasing the risk of early school leaving (Pérez-Marco et al., 2026b). From this perspective, educational guidelines and policies should promote a systemic reconfiguration of the school environment, transforming it from a potential risk factor into a protective resource. From a strictly educational standpoint, this means moving beyond purely summative and comparative assessment practices in favor of a formative approach—one focused on mastering skills—that can highlight the incremental progress of individual students (Martínez-Maireles et al., 2022). On a psycho-pedagogical level, adopting a formative approach centered on collaborative learning design and continuous feedback fundamentally redefines the nature of error. By replacing summative grading with competence-oriented guidance, mistakes cease to be a final verdict and instead become actionable operational data (Carless & Boud, 2018; Andrade, 2019). This paradigm shifts the student’s focus toward controllable variables, fostering a growth mindset and dismantling the belief that success depends on immutable, innate factors. Within this framework, the objective validation of micro-incremental progress restructures cognitive self-efficacy patterns, and defuses the mechanisms of LH, ultimately restoring intrinsic motivation and behavioral self-regulation processes (Andrade, 2019; Carless & Boud, 2018). Therefore, the systematic implementation of formative approach is essential to ensuring optimal success thereby deconstructing the stable, global causal attributions that fuel LH.

However, interventions targeting teaching practices must be intrinsically embedded within the student–teacher relationship (Kraft et al., 2018). To this end, targeted initiatives for continuous teacher professional development should not only equip educators with the knowledge and skills needed to recognize early warning signs of student disengagement but also foster the emotional and relational competencies required to co-create a classroom climate grounded in psychological safety (Pianta et al., 2008). These competencies should be understood as a set of resources that can support educators in responding flexibly to students’ needs, while also considering the broader relational and contextual factors that may contribute to school attendance problems. Within such an environment, students are not only welcomed, valued, and understood, but are also encouraged by their teachers to perceive learning as a developmental process characterized by both achievements and setbacks. In this context, perseverance and self-efficacy become fundamental, while mistakes are reframed as a constructive and indispensable component of learning (Davis et al., 2019). From this perspective, the educational guidelines proposed in the present study also emphasize the importance of structured, evidence-based, and continuous teacher professional development throughout the entire teaching career. Rather than merely transmitting theoretical knowledge, these initiatives should provide practical strategies, reflective supervision, and opportunities for collaborative learning (Davis et al., 2019; Valkov & Lavrentsova, 2019). Such an approach enables educators to effectively safeguard students’ psychological well-being while simultaneously equipping them with strategies to enhance school engagement and active participation. Equally important, professional development should cultivate teachers’ own emotional competence, resilience, and self-awareness, enabling them to navigate the complex relational dynamics of the classroom and proactively manage challenges such as prolonged student absenteeism. By investing in sustained teacher professional development, educational systems can transform the promotion of mental health from an isolated intervention into a permanent and integrated component of everyday pedagogy, ultimately fostering learning environments in which both students and teachers can thrive.

Taken together, the findings of the present study suggest that educational guidelines should proactively address the ecological and situational factors underlying school disengagement rather than merely responding to its symptomatic and externalized manifestations, such as school absenteeism (Kearney et al., 2023b; Sälzer et al., 2024). This principle constitutes the central premise of the educational guidelines presented in this study, which seek to provide an authentic, systemic, and evidence-informed strategy for preventing early school leaving, with important implications for multiple stakeholders (Sälzer et al., 2024). For educators and school staff, these guidelines offer evidence-based recommendations for designing and adapting teaching practices that promote psychologically safe, supportive, and inclusive learning environments, enabling them to identify and address early warning signs before they escalate into chronic absenteeism. For school leaders and policymakers, the findings and educational guidelines underscore the urgent need to invest in sustained teacher professional development and to allocate resources toward systemic screening and monitoring protocols capable of identifying early risk factors. By implementing data-informed monitoring systems alongside interventions that simultaneously strengthen student well-being and teachers’ professional capacity, educational leaders can institutionalize a preventive rather than reactive approach to school disengagement. For school psychologists and counselors, the present study and educational guidelines highlight the need for a more systemic and preventive framework. Their role should increasingly involve implementing school-wide screening protocols, identifying early indicators of risk, and delivering targeted, evidence-based interventions. At the same time, they serve as essential consultants for teachers and families, facilitating interdisciplinary collaboration aimed at strengthening students’ emotional resilience, reinforcing their sense of school belonging, and preventing the escalation of disengagement. Finally, for the scientific community, this study identifies several priorities for future research. In particular, it calls for longitudinal and intervention-based studies to clarify the influence of psychological vulnerability, classroom climate, teacher–student relationships and pedagogical practices on school engagement, and their potential contribution to school absenteeism and ultimately early school leaving.

## 6. Conclusions

Overall, the study’s findings suggest the existence of a potential reciprocal relationship between SAPs and LH. Gradual school absenteeism may be a consequence of initial difficulties, as well as a condition that contributes to their persistence over time, through a process of mutual reinforcement among the variables considered. This study therefore underscores the importance of emotional, motivational, and cognitive vulnerabilities, perceived competence, and the school context in understanding SAP. It highlights how these factors may be associated with patterns of school attendance difficulties over time. These findings provide valuable insights into breaking this dysfunctional reciprocal process between SAPs and LH and promoting it into a virtuous cycle through the application of specific educational guidelines.

## 7. Limitation

Although this study has shed light on the role of certain individual and contextual factors as risk and protective factors, as well as potential consequences of SAPs, it has some limitations. Firstly, the analyzed data pertain to Italian adolescent students, which limits the generalizability of the results to other cultural contexts and age groups. Therefore, future studies should explore these relationships within different educational and cultural systems.

Secondly, a moderate dropout rate was observed among participants during the longitudinal surveys due to various reasons, such as absences, school transfers and difficulties in maintaining contact over time. This is a phenomenon that is consistent with previous research conducted in longitudinal educational settings (Bianchi et al., 2022; Henneberger et al., 2023). As the study looked at problems with school attendance, we can guess that the loss of people taking part may have affected the final group of students in the study. In particular, students experiencing more severe attendance difficulties may have been less likely to complete the study. Although the extent of this potential bias cannot be determined, it should be considered when interpreting the findings. Therefore, future studies should place greater emphasis on promoting adherence and participation throughout the various phases of the research, including the use of more flexible methods for administering self-report questionnaires.

Another limitation of this study is that it relied exclusively on self-report measures to investigate all the variables under consideration. Using self-assessment questionnaires completed by students could introduce biases related to the participants’ subjective perceptions (Fernández-Montalvo & Echeburúa, 2006). Therefore, future studies should incorporate various assessment tools, such as interviews, behavioral observations and data from the school setting (Kearney & Albano, 2018; Kearney & Silverman, 1993).

Furthermore, this study is based on a cross-lagged panel model with only two time points. While this approach allows the examination of reciprocal longitudinal associations among the variables under investigation, the methodological literature indicates that at least three waves are needed to better capture dynamic and intra-individual processes over time (Hamaker et al., 2015; Orth et al., 2021). In addition, although observed variables were used in the analyses, longitudinal measurement invariance was not tested; future research should address this issue to ensure construct comparability across time. Moreover, although theoretically sound, the use of a traditional CLPM with a relatively high number of observed variables and only two waves may limit causal interpretations of the associations between individual and contextual factors related to SAPs. Accordingly, future studies should replicate these findings using alternative longitudinal approaches, such as the random intercept cross-lagged panel model (RI-CLPM) and include additional waves of data collection to provide a more robust understanding of developmental trajectories and a more accurate interpretation of the observed associations.

## Figures and Tables

**Table 1:** Descriptive Statistics and internal consistency for Study Variables.

					Skewness		Kurtosis	
	Means	SD	Min	Max	Skewness	SE	Kurtosis	SE
SAPs T1	0.70	0.45	0.00	2.15	0.70	0.10	−0.01	0.21
General Competence T1	4.60	1.45	1.00	7.00	−0.34	0.10	−0.76	0.21
Academic Competence T1	4.95	1.31	1.25	7.00	−0.48	0.10	−0.29	0.21
Relational Competence T1	5.08	1.36	1.00	7.00	−0.63	0.10	−0.15	0.21
Leisure Competence T1	4.84	1.19	1.50	7.00	−0.24	0.10	−0.46	0.21
Academic Procrastination T1	2.79	1.09	1.00	5.00	0.21	0.10	-0.93	0.21
Mastery Orientation T1	3.54	0.69	1.86	5.00	−0.14	0.10	−0.34	0.21
Learned Helplessness T1	2.69	0.99	1.00	5.00	0.34	0.10	−0.69	0.21
Teacher Support T1	3.00	0.86	1.25	5.00	0.13	0.10	−0.58	0.21
Peer Connection T1	3.73	0.80	1.00	5.00	−0.59	0.10	0.09	0.21
School Connection T1	3.31	0.83	1.00	5.00	−0.14	0.10	−0.37	0.21
Affirming Diversity T1	3.77	0.86	1.00	5.00	−0.75	0.10	0.24	0.21
Clarity of Rules T1	3.78	0.80	1.00	5.00	−0.41	0.10	−0.40	0.21
Reporting and Seeking Help T1	3.56	1.02	1.00	5.00	−0.38	0.10	−0.68	0.21
SAPs T2	0.77	0.47	0.00	2.31	0.62	0.10	0.03	0.21
General Competence T2	4.59	1.40	1.00	7.00	−0.29	0.10	−0.65	0.21
Academic Competence T2	4.85	1.30	1.25	7.00	−0.42	0.10	−0.38	0.21
Relational Competence T2	4.92	1.35	1.00	7.00	−0.40	0.10	−0.55	0.21
Leisure Competence T2	4.84	1.12	1.75	7.00	−0.05	0.10	−0.49	0.21
Academic Procrastination T2	3.01	1.04	1.00	5.00	0.00	0.10	−0.78	0.21
Mastery Orientation T2	3.39	0.73	1.43	5.00	−0.04	0.10	−0.49	0.21
Learned Helplessness T2	2.76	0.93	1.00	5.00	0.17	0.10	−0.54	0.21
Teacher Support T2	3.09	0.83	1.00	5.00	0.02	0.10	−0.48	0.21
Peer Connection T2	3.62	0.79	1.00	5.00	−0.33	0.10	−0.23	0.21
School Connection T2	3.24	0.78	1.00	5.00	−0.01	0.10	−0.26	0.21
Affirming Diversity T2	3.71	0.84	1.00	5.00	−0.34	0.10	−0.56	0.21
Clarity of Rules T2	3.57	0.82	1.00	5.00	−0.10	0.10	−0.47	0.21
Reporting and Seeking Help T2	3.43	0.92	1.00	5.00	−0.17	0.10	−0.41	0.21

Note: T1 = Time 1; T2 = Time 2. The sample comprised 523 participants at both T1 and T2 (nT1 = 523; nT2 = 523).

**Table 2:** Correlational analyses for Study Variables.

	1	2	3	4	5	6	7	8	9	10	11	12	13	14	15	16	17	18	19	20	21	22	23	24	25	26	27	28
1. SAPs (T1)	—																											
2. General Competence (T1)	−0.28 ***	—																										
3. Academic Competence (T1)	−0.28 ***	0.44 ***	—																									
4. Relational Competence (T1)	−0.07	0.41 ***	0.22 ***	—																								
5. Leisure Competence (T1)	−0.17 ***	0.48 ***	0.33 ***	0.49 ***	—																							
6. Academic Procrastination (T1)	0.36 ***	−0.25 ***	−0.45 ***	−0.09 *	−0.22 ***	—																						
7. Mastery Orientation (T1)	−0.19 ***	0.14 **	0.46 ***	0.15 ***	0.29 ***	−0.45 ***	—																					
8. Learned Helplessness (T1)	0.29 ***	−0.58 ***	−0.48 ***	−0.35 ***	−0.38 ***	0.26 ***	−0.19 ***	—																				
9. Teacher Support (T1)	−0.18 ***	0.12 **	0.27 ***	0.13 **	0.11 **	−0.29 ***	0.31 ***	−0.13 **	—																			
10. Peer Connectedness (T1)	−0.14 **	0.29 ***	0.13 **	0.64 ***	0.29 ***	−0.08	0.15 ***	−0.28 ***	0.21 ***	—																		
11. School Connectedness (T1)	−0.23 ***	0.34 ***	0.39 ***	0.40 ***	0.25 ***	−0.27 ***	0.32 ***	−0.35 ***	0.59 ***	0.56 ***	—																	
12. Affirming Diversity (T1)	−0.15 ***	0.12 **	0.27 ***	0.21 ***	0.16 ***	−0.18 ***	0.25 ***	−0.16 ***	0.39 ***	0.36 ***	0.57 ***	—																
13. Rule Clarity (T1)	−0.18 ***	0.09 *	0.21 ***	0.15 ***	0.13 **	−0.30 ***	0.26 ***	−0.11 **	0.48 ***	0.27 ***	0.54 ***	0.54 ***	—															
14. Reporting and Seeking Help (T1)	−0.17 ***	0.17 ***	0.21 ***	0.22 ***	0.19 ***	−0.22 ***	0.20 ***	−0.16 ***	0.56 ***	0.33 ***	0.57 ***	0.49 ***	0.65 ***	—														
15. SAPs (T2)	0.59 ***	−0.27 ***	−0.33 ***	−0.07	−0.16 ***	0.30 ***	−0.21 ***	0.35 ***	−0.14 **	−0.12 **	−0.23 ***	−0.15 ***	−0.10 *	−0.10 *	—													
16. General Competence (T2)	−0.15 ***	0.61 ***	0.34 ***	0.31 ***	0.42 ***	−0.19 ***	0.12 **	−0.46 ***	0.10 *	0.21 ***	0.22 ***	0.04	0.05	0.12 **	−0.26 ***	—												
17. Academic Competence (T2)	−0.24 ***	0.34 ***	0.69 ***	0.14 **	0.26 ***	−0.35 ***	0.39 ***	−0.43 ***	0.16 ***	0.11 **	0.32 ***	0.22 ***	0.14 **	0.16 ***	−0.36 ***	0.47 ***	—											
18. Relational Competence (T2)	−0.08	0.37 ***	0.19 ***	0.66 ***	0.39 ***	−0.09 *	0.16 ***	−0.32 ***	0.03	0.48 ***	0.29 ***	0.13 **	0.15 ***	0.13 **	−0.10 *	0.39 ***	0.25 ***	—										
19. Leisure Competence (T2)	−0.15 ***	0.36 ***	0.22 ***	0.37 ***	0.56 ***	−0.16 ***	0.18 ***	−0.28 ***	0.03	0.19 ***	0.15 ***	0.08 *	0.09 *	0.10 *	−0.16 ***	0.49 ***	0.32 ***	0.53 ***	—									
20. Academic Procrastination (T2)	0.33 ***	−0.18 ***	−0.37 ***	−0.03	−0.08 *	0.66 ***	−0.34 ***	0.25 ***	−0.25 ***	−0.00	−0.20 ***	−0.12 **	−0.20 ***	−0.18 ***	0.41 ***	−0.25 ***	−0.46 ***	−0.04	−0.10 *	—								
21. Mastery Orientation (T2)	−0.14 ***	0.08 *	0.38 ***	0.04	0.14 ***	−0.33 ***	0.60 ***	−0.20 ***	0.18 ***	0.05	0.21 ***	0.18 **	0.7 ***	0.10 *	−0.25 ***	0.09 *	0.50 ***	0.08	0.19 **	−0.41 ***	—							
22. Learned Helplessness (T2)	0.23 ***	−0.49 ***	−0.41 ***	−0.22 ***	−0.36 ***	0.15 ***	−0.19 ***	0.67 ***	−0.10 *	−0.18 ***	−0.27 ***	−0.08 *	−0.06	−0.12 **	0.37 ***	−0.60 ***	−0.54 ***	−0.3 ***	−0.37 ***	0.28 ***	−0.18 ***	—						
23. Teacher Support (T2)	−0.16 ***	0.12 **	0.24 ***	0.01	0.07	−0.23 ***	0.24 ***	−0.18 ***	0.51 ***	0.07	0.39 ***	0.27 ***	0.36 ***	0.39 ***	−0.22 ***	0.13 **	0.31 ***	0.07	0.12 **	−0.34 ***	0.34 **	−0.16 ***	—					
24. Peer Connectedness (T2)	−0.13 **	0.22 ***	0.13 **	0.39 ***	0.26 ***	−0.11 **	0.21 ***	−0.24 ***	0.11 **	0.48 ***	0.28 ***	0.25 ***	0.19 ***	0.14 ***	−0.14 **	0.24 ***	0.22 ***	0.54 ***	0.33 ***	−0.08 *	0.23 ***	−0.24 ***	0.25 ***	—				
25. School Connectedness (T2)	−0.21 ***	0.27 ***	0.28 ***	0.25 ***	0.19 ***	−0.22 ***	0.28 ***	−0.26 ***	0.36 ***	0.30 ***	0.50 ***	0.33 ***	0.38 ***	0.35 ***	−0.26 ***	0.27 ***	0.38 ***	0.35 ***	0.26 ***	−0.30 ***	0.37 ***	−0.27 ***	0.62 ***	0.57 ***	—			
26. Affirming Diversity (T2)	−0.17 ***	0.05	0.23 ***	0.05	0.10 *	−0.14 **	0.26 ***	−0.11 **	0.20 ***	0.15 ***	0.24 ***	0.48 ***	0.35 ***	0.24 ***	−0.22 ***	0.07	0.28 ***	0.14 ***	0.19 ***	−0.17 ***	31 ***	−0.11 **	0.37 ***	0.43 ***	0.52 ***	—		
27. Rule Clarity (T2)	−0.19 ***	0.08	0.20 ***	0.05	0.07	−0.24 ***	0.26 ***	−0.04	0.32 ***	0.09 *	0.32 ***	0.31 ***	0.54 ***	0.41 ***	−0.15 ***	0.06	0.25 ***	0.09 *	15 ***	−0.27 *	0.33 ***	−0.03	0.54 ***	0.33 **	0.60 ***	0.53 ***	—	
28. Reporting and Seeking Help (T2)	−0.15 ***	0.15 ***	0.16 ***	0.14 **	0.12 *	−0.18 ***	0.22 ***	−0.07	0.39 ***	0.18 ***	0.37 ***	0.33 ***	0.43 ***	0.54 ***	−0.15 ***	0.13 **	0.23 ***	0.16 ***	0.14 ***	−0.23 **	0.26 ***	−0.09 *	0.60 ***	0.33 ***	0.60 ***	0.48 ***	0.66 ***	—

Note. 1 = SAPs (T1); 2 = General Competence (T1); 3 = Academic Competence (T1); 4 = Relational Competence (T1); 5 = Leisure Competence (T1); 6 = Academic Procrastination (T1); 7 = Mastery Orientation (T1); 8 = Learned Helplessness (T1); 9 = Teacher Support (T1); 10 = Peer Connectedness (T1); 11 = School Connectedness (T1); 12 = Affirming Diversity (T1); 13 = Rule Clarity (T1); 14 = Reporting and Seeking Help (T1); 15 = SAPs (T2); 16 = General Competence (T2); 17 = Academic Competence (T2); 18 = Relational Competence (T2); 19 = Leisure Competence (T2); 20 = Academic Procrastination (T2); 21 = Mastery Orientation (T2); 22 = Learned Helplessness (T2); 23 = Teacher Support (T2); 24 = Peer Connectedness (T2); 25 = School Connectedness (T2); 26 = Affirming Diversity (T2); 27 = Rule Clarity (T2); 28 = Reporting and Seeking Help (T2). * *p* < 0.05, ** *p* < 0.01, *** *p* < 0.001.

## Data Availability

The datasets generated and/or analyzed during the current study are not publicly available due to privacy and ethical restrictions but are available from the University of Messina (Prof. Luana Sorrenti) upon reasonable request.
